# Supplementary material for: From sequence to enzyme mechanism using multi-label machine learning
Source: BMC Bioinformatics. 2014 May 19;15:150. doi: 10.1186/1471-2105-15-150 (PMC4229970; doi:10.1186/1471-2105-15-150)
Supplement: Additional file 2 — Java code of ml2db. Additional file ml2db_code.tar.gz contains the Java source code to run the multi-label machine learning experiments and save the results to database. The code’s Javadoc is included. [file 1471-2105-15-150-S2.zip › additional file 2/ml2db/ml2db/doc/main/ml2db/mulan/mulanarff/package-tree.html]

main.ml2db.mulan.mulanarff Class Hierarchy


JavaScript is disabled on your browser.


- Overview
- Package
- Class
- Use
- Tree
- Deprecated
- Index
- Help

- Prev
- Next

- Frames
- No Frames

- All Classes

# Hierarchy For Package main.ml2db.mulan.mulanarff

Package Hierarchies:

- All Packages

## Class Hierarchy

- java.lang.Object
  - weka.core.AbstractInstance (implements weka.core.Instance, weka.core.RevisionHandler, java.io.Serializable)
    - weka.core.SparseInstance
      - main.ml2db.mulan.mulanarff.IdInstance (implements java.lang.Comparable<T>)
  - main.ml2db.weka.AttributeFactory
    - main.ml2db.mulan.mulanarff.MulanAttributeFactory
  - main.ml2db.mulan.mulanarff.AttributeUtils
  - main.ml2db.weka.DataSetDbLoader
    - main.ml2db.mulan.mulanarff.MulanDataSetDbLoader
  - main.ml2db.weka.DataSetGenerator
    - main.ml2db.mulan.mulanarff.MulanDataSetGenerator
  - main.ml2db.weka.ImportArff
    - main.ml2db.mulan.mulanarff.ImportMulanArff
  - uk.ac.standrews.utils.main.Initialised (implements uk.ac.standrews.utils.main.Initialisable)
    - main.ml2db.weka.Arff
      - main.ml2db.mulan.mulanarff.MulanArff
    - main.ml2db.weka.ArffProps
      - main.ml2db.weka.ArffPropsFromFile
        - main.ml2db.mulan.mulanarff.MulanArffProperties
  - main.ml2db.weka.InstancesFiller
    - main.ml2db.mulan.mulanarff.MulanInstancesFiller
  - main.ml2db.mulan.mulanarff.InstanceUtils
  - uk.ac.standrews.utils.main.database.Manager
    - main.ml2db.weka.DataSetManager
      - main.ml2db.mulan.mulanarff.MulanDataSetManager
  - mulan.data.MultiLabelInstances (implements java.io.Serializable)
    - main.ml2db.mulan.mulanarff.IdMultiLabelInstances (implements java.io.Serializable)
  - main.ml2db.mulan.mulanarff.NeighboursPrinter
  - main.ml2db.mulan.mulanarff.NeighboursPrinterTest
  - main.ml2db.mulan.mulanarff.Statistics (implements java.io.Serializable)

- Overview
- Package
- Class
- Use
- Tree
- Deprecated
- Index
- Help

- Prev
- Next

- Frames
- No Frames

- All Classes
